# A Simple Technique for Deploying the SENTINEL Cerebral Protection System in Bovine Aortic Arch Anatomy

**DOI:** 10.1016/j.shj.2023.100228

**Published:** 2023-10-16

**Authors:** Ajoe John Kattoor, Christopher Manion, Vijay Iyer

**Affiliations:** Division of Structural Cardiology, University at Buffalo, Kaleida Health, Gates Vascular Institute, Buffalo, New York, USA

**Keywords:** Bovine arch embolic protection, Sentinel embolic protection, Sentinel in bovine arch

## Abstract

The SENTINEL Cerebral Protection System is one of the most commonly used devices for embolic protection during transcatheter aortic valve replacement. However, successful deployment of the SENTINEL device is often challenging in patients with a bovine aortic arch anatomy using the standard technique and requires extensive manipulation in the aortic arch increasing the risk of stroke. We describe a novel and simple technique of 2-filter deployment of SENTINEL device in patients with bovine arch anatomy. In this technique, after the deployment of the proximal filter, the device is hyperflexed on itself facing the lateral aspect of the ascending aorta instead of facing the descending aorta, with its tip pointing toward the common origin of the left common carotid artery (LCCA) and brachiocephalic trunk. The guidewire is then advanced to the LCCA. Since the guidewire can pass either anterior or posterior to the device shaft, the device needs to be untwisted either by clockwise or counterclockwise motion, before pulling the device shaft back to engage the LCCA, after which the distal filter can be deployed. Computed tomography scans obtained for planning transcatheter aortic valve replacement should be reviewed for the presence of bovine aortic arch anatomy so that this technique can be deployed directly, thereby reducing manipulations in the aortic arch, saving time, and not requiring additional equipment.

## Background

**Figure 1:**
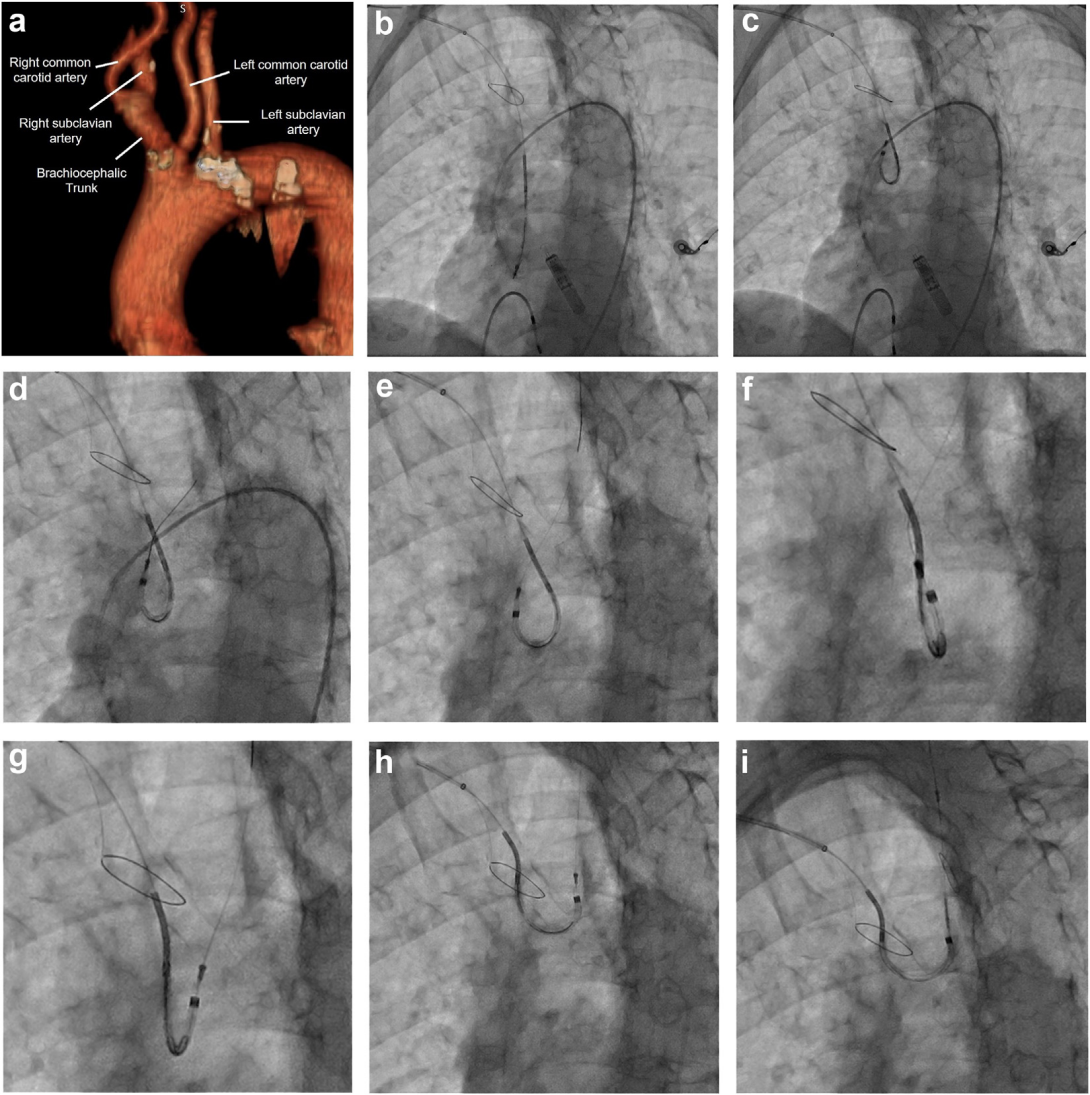
(a) Volume rendered computed tomographic image depicting bovine aortic arch anatomy; (b) The SENTINEL Cerebral Protection System is positioned in the usual fashion, at the junction of the brachiocephalic trunk (BCT) and the ascending aorta, with the proximal filter deployed in the BCT; (c) The device is hyperflexed on itself facing the lateral aspect of the ascending aorta, to point towards the common origin of the left common carotid artery (LCCA) and the BCT; (d and e) The GRAND SLAM guidewire is advanced into the LCCA; (f) Application of gentle clockwise rotation while the device is still positioned in the aortic arch, but resulting in twisting and mild resistance; (g) Subsequent application of counterclockwise rotation untwisting the device; (h) Subsequent further application of counterclockwise rotation followed by a pull-back to engage the LCCA; (i) Successful distal filter deployment in the LCCA.

The SENTINEL Cerebral Protection System (Boston Scientific; Marlborough, MA) is one of the most commonly used and widely studied devices for embolic protection during transcatheter aortic valve replacement (TAVR).^1^ Successful deployment of the SENTINEL device is often challenging in patients with a bovine aortic arch (BAA) anatomy using the standard technique. In the PROTECTED-TAVR trial, 94.4% of patients who were randomized had a successful device deployment.^2^ However, patients with bovine arch anatomy were not included in the study, as they were considered to have unfavorable anatomy. In an unselected patient group, and among low-volume operators, successful two-filter deployment of the SENTINEL device may be lower. In a single-center study of patients undergoing TAVR, Tagliari et al. reported the prevalence of BAA at 12% and successful two-filter deployment was relatively low in patients with BAA (BAA vs non-BAA, 65 vs 89%; *p* = 0.002).^3^ Placement of coronary guidewire in the left common carotid artery (LCCA) using a JR4 diagnostic catheter, followed by delivery of the SENTINEL device has been previously described with a reported 100% deployment success rate in BAA anatomy.^4^ Here, we describe another simple method for SENTINEL device delivery in patients found to have BAA anatomy (Figure 1).

## Description

A 0.014” GRAND SLAM guidewire (Asahi Intecc Medical; Irvine, CA) is advanced into the ascending aorta from the right radial artery. The SENTINEL Cerebral Protection System is then advanced over the guidewire and positioned in the usual fashion, at the junction of the brachiocephalic trunk (BCT) and the ascending aorta. The proximal filter of the device is then deployed in the BCT. The shaft of a pigtail catheter placed in the aortic root is used to delineate the greater curvature of the aortic arch during this step. The GRAND SLAM guidewire is then pulled back into the SENTINEL device and the distal portion of the device is flexed concurrently with partial pull-back into the aortic arch. However, unlike the usual clockwise rotation of device to face the descending aorta, the device is hyperflexed on itself facing the lateral aspect of the ascending aorta, to point towards the common origin of the LCCA and the BCT, as shown in Figure 1. Thus, the device now points in the direction of the LCCA in a patient with BAA anatomy. The GRAND SLAM guidewire can then be advanced into the LCCA. The guidewire has now passed either anterior or posterior to the shaft of the SENTINEL device. In this position, a gentle clockwise rotation is made while the device is still positioned in the aortic arch. If the guidewire is anterior to the shaft of the device, then the device will untwist and can be further pulled back to engage the LCCA. However, if there is mild resistance and the device appears to start twisting around the guidewire on the fluoroscopy, the guidewire has passed posterior to the shaft of the device (Figure 1e). Therefore, a counterclockwise rotation must be made to untwist the device followed by a gentle pull-back to engage the LCCA. The distal filter can then be deployed in the usual fashion. Of note, after obtaining guidewire position in the LCCA, it is best to withdraw any other hardware (such as the pigtail catheter) from the aortic arch, to avoid entanglement during distal filter deployment.

## Discussion

Engagement of the LCCA for two-filter deployment in patients with BAA anatomy often requires extensive device manipulation in the aortic arch which can increase the risk of stroke. We have had repeated success in patients with BAA anatomy as evidenced by successful two-filter deployment on multiple consecutive patients where we used this technique. Only patients who had a common origin of BCT and LCCA (as in Figure 1a) with appropriately sized vessels and no proximal atherosclerotic disease on review of the TAVR-planning computed tomography (CT) received this technique. All cases were performed with minimal manipulations and no adverse events (Supplemental Video 1). CT scans obtained for TAVR planning should be reviewed for the presence of BAA anatomy so that this technique can be deployed directly, thereby reducing manipulations in the aortic arch, saving time, and not requiring a JR4 catheter.

## Consent Statement

The research reported has adhered to the relevant ethical guidelines and the patient signed an informed procedural consent.

## Funding

The authors have no funding to report.

## Disclosure Statement

Vijay Iyer reports a relationship with Boston Scientific Corp that includes consulting or advisory and with Edwards Lifesciences Corporation that includes speaking and lecture fees. All other authors report no conflicts of interest.

## Supplementary Material

Supplemental Video 1 Depicts Sentinel Cerebral Embolic Protection System deployment in a bovine aortic arch anatomy using the described technique.
